# Assessment of ^18^F-DCFPyL PSMA PET/CT and PET/MR quantitative parameters for reference standard organs: Inter-reader, inter-modality, and inter-patient variability

**DOI:** 10.1371/journal.pone.0283830

**Published:** 2023-04-06

**Authors:** Edward M. Lawrence, Minnie Kieler, Greg Cooley, Shane A. Wells, Steve Y. Cho

**Affiliations:** 1 Department of Radiology, University of Wisconsin-Madison, Madison, Wisconsin, United States of America; 2 Department of Human Oncology, University of Wisconsin-Madison, Madison, Wisconsin, United States of America; 3 Carbone Cancer Center, Madison, Wisconsin, United States of America; IRCCS Ospedale Policlinico San Martino, Genova, Italy, ITALY

## Abstract

Prostate specific membrane antigen (PSMA)-based radiotracers have shown promise for prostate cancer assessment. Evaluation of quantitative variability and establishment of reference standards are important for optimal clinical and research utility. This work evaluates the variability of PSMA-based [^18^F]DCFPyL (PyL) PET quantitative reference standards. Consecutive eligible patients with biochemically recurrent prostate cancer were recruited for study participation from August 2016-October 2017. After PyL tracer injection, whole body PET/CT (wbPET/CT) was obtained with subsequent whole body PET/MR (wbPET/MR). Two readers independently created regions of interest (ROIs) including a 40% standardized uptake value (SUV) threshold ROI of the whole right parotid gland and separate spherical ROIs in the superior, mid, and inferior gland. Additional liver (right lobe) and blood pool spherical ROIs were defined. Bland-Altman analysis, including limits of agreement (LOA), as well as interquartile range (IQR) and coefficient of variance (CoV) was used. Twelve patients with prostate cancer were recruited (mean age, 61.8 yrs; range 54–72 years). One patient did not have wbPET/MR and was excluded. There was minimal inter-reader SUV_mean_ variability (bias±LOA) for blood pool (-0.13±0.42; 0.01±0.41), liver (-0.55±0.82; -0.22±1.3), or whole parotid gland (-0.05±0.31; 0.08±0.24) for wbPET/CT and wbPET/MR, respectively. Greater inter-reader variability for the 1-cm parotid gland ROIs was present, for both wbPET/CT and wbPET/MR. Comparing wbPET/CT to the subsequently acquired wbPET/MR, blood pool had a slight decrease in SUV_mean_. The liver as well as parotid gland showed a slight increase in activity although the absolute bias only ranged from 0.45–1.28. The magnitude of inter-subject variability was higher for the parotid gland regardless of modality or reader. In conclusion, liver, blood pool, and whole parotid gland quantitation show promise as reliable reference normal organs for clinical/research PET applications. Variability with 1-cm parotid ROIs may limit its use.

## Introduction

Prostate cancer is a common malignancy [1] and accurate staging is important for treatment planning and response evaluation [2, 3]. Unfortunately, traditional imaging (i.e. CT, pelvic MR, bone scan, and [^18^F]FDG PET) has limited accuracy for staging and detecting disease recurrence [3–6].

PSMA-based PET has shown promise in this setting [7–22]. In addition to lesion detection, PSMA expression has been correlated with Gleason score [23, 24] and increased PSMA expression in a suspicious lesion has been correlated with increased likelihood of true metastatic disease [25]. Evaluating changes in PSMA tracer expression may also be useful in evaluating treatment response [21, 22]. Much of the prior work with PSMA-based tracers has focused on PET/CT acquisitions with more limited evaluation of hybrid PET/MR [10, 14]. In particular, further investigation into the [^18^F]DCFPyL tracer is especially timely given its recent Food and Drug Administration approval.

Common or agreed upon reader interpretation guidelines for PSMA-based PET are needed. Prior publications, including the recently published E-PSMA standardized reporting guidelines [26], have included quantitative reference organs that might be used; however, evaluation of the variability and reproducibility of these reference standards has been more limited [26–31]. Therefore, the purpose of this work was to evaluate the variability of PSMA-based [^18^F]DCFPyL (PyL) PET quantitative reference standards.

## Materials and methods

This prospective observational study was approved by the University of Wisconsin-Madison Institutional Review Board and maintained full compliance with the Health Insurance Portability and Accountability Act.

### Patients

From August 2016 –October 2017, consecutive eligible subjects were recruited for enrollment in the study. Subjects were eligible for inclusion if they (1) had a history of prostate cancer with prior radical prostatectomy, (2) had current evidence of biochemical recurrence with plan for salvage external-beam radiation therapy with or without androgen deprivation therapy, and (3) could undergo MRI. Patients were excluded if (1) they had a history of prior radiation therapy, chemotherapy, or androgen deprivation therapy for prostate cancer or (2) if they had a history of any other malignancy within the last 2 years, other than skin basal cell or cutaneous superficial squamous cell carcinoma that has not metastasized and superficial bladder cancer. Written informed consent was obtained from all participants.

### Image acquisition technique

[^18^F]DCFPyL-PSMA tracer was injected with a median dose of 7.44 mCi (range 6.03–8.82) [275.28 MBq (range 223.11–326.34 MBq)]. After injection a whole body (wb) PET/CT (Discovery 710 PET/CT, GE Healthcare, Waukesha, WI) was immediately followed by a wbPET/MR (Signa PET/MR, GE Healthcare, Waukesha, WI). Acquisition parameters are listed in Table 1.

**Table 1:** PET acquisition parameters.

	wbPET/CT	wbPET/MR
Scanner	GE Discovery 710	GE Signa
PET timing (after PyL injection)	~ 60 minutes	~ 120 minutes
Acquisition/Reconstruction	3D with OSEM	3D with OSEM
Matrix	192 x 192	192 x 192
Iterations/Subsets	3/24	3/28
Time per bed position	3 min	3 min

GE, General Electric; wb, whole body; PET, positron emission tomography; CT, computed tomography; MR, magnetic resonance.

### Region of interest (ROI) generation

Two board-certified readers (4 and 6 years of experience in molecular imaging, E.M.L. and M.L. respectively) independently reviewed the PET/CT and PET/MR data and created volumetric ROIs using Mirada XD software (Oxford, UK). A 40% standardized uptake value (SUV) threshold method was used to define the whole right parotid gland. In addition, 1-cm spherical ROIs were placed in the superior, mid, and inferior parotid gland. Finally, a 3-cm spherical ROI was used to assess hepatic uptake (using the right hepatic lobe) and a 1-cm spherical ROI was used to assess blood pool (using the descending aorta). For the quantitative analysis included in the current study, prostate cancer related lesions were not considered. This choice was made in part because multiple subjects did not have confirmed sites of PSMA+ recurrent disease.

### Statistical analysis

SUV_mean_ was used for comparison. Bland-Altman plots, including calculation of bias and limits of agreement (LOA), were used and interquartile range (IQR) was assessed [32]. The coefficient of variance (CoV) was calculated by dividing the standard deviation by the population mean and multiplying the result by 100. The data was collated using Microsoft Excel (v. 2010, Microsoft, Redmond, WA) and additional statistical analysis, including Bland-Altman analysis, was performed using Matlab (MathWorks, Natick, MA).

## Results

Twelve patients were recruited (mean age, 61.8 yrs; range 54–72 years). One patient did not complete the wbPET/MR and was therefore excluded.

### Inter-reader variability

**Fig 1:**
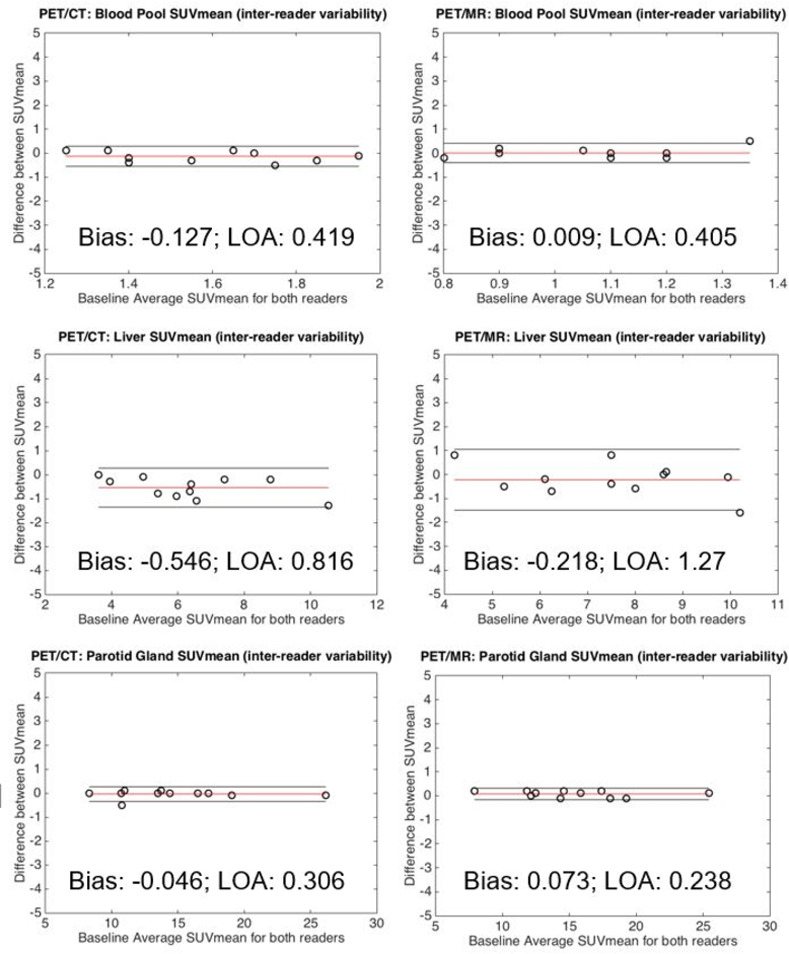
Bland Altman analysis assessing inter-reader variability in blood pool, liver, and whole parotid gland. Minimal inter-reader SUV_mean_ variability is seen for blood pool (top), liver (middle), and whole parotid gland (bottom) for both wbPET/CT (left column) and wbPET/MR (right column). The calculated bias (red line) as well as the upper and lower limits of agreement (outer black lines) are demarcated on each plot. The bias and limits of agreement are listed in the top-center or bottom-center of each plot.

**Fig 2:**
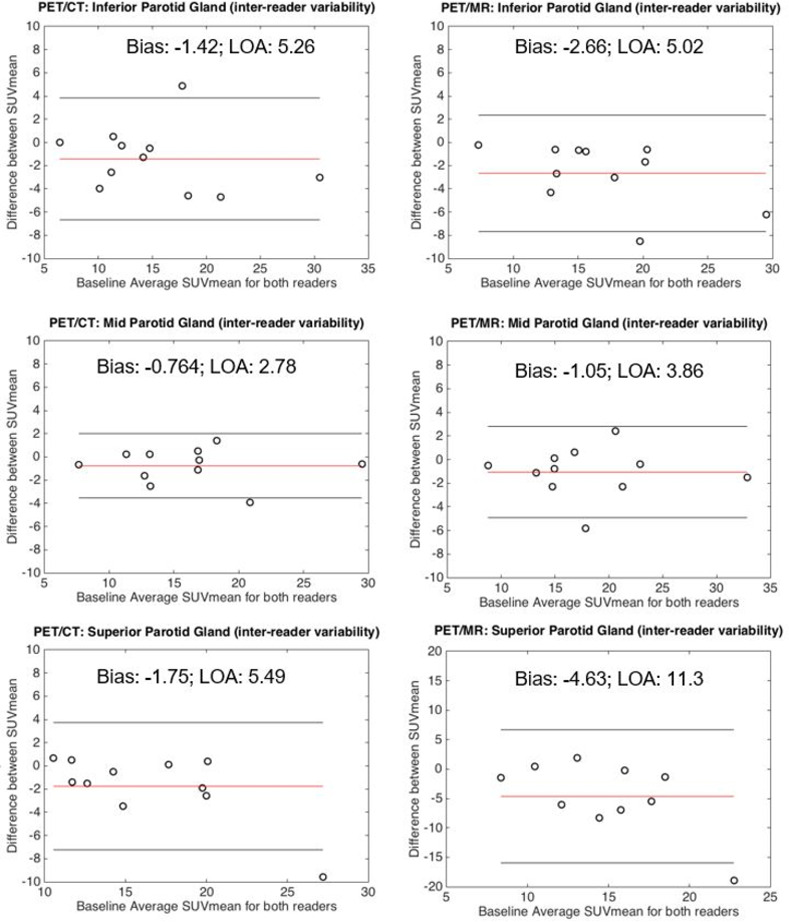
Bland Altman analysis assessing inter-reader variability for 1-cm parotid regional regions of interest. Greater bias and limits of agreement were seen for the 1-cm parotid circular ROIs in the inferior (top), mid (middle), or superior (bottom) parotid gland for both wbPET/CT (left column) or wbPET/MR (right column). The calculated bias (red line) as well as the upper and lower limits of agreement (outer black lines) are demarcated on each plot. The bias and limits of agreement are listed in the top-center or bottom-center of each plot.

**Fig 3:**
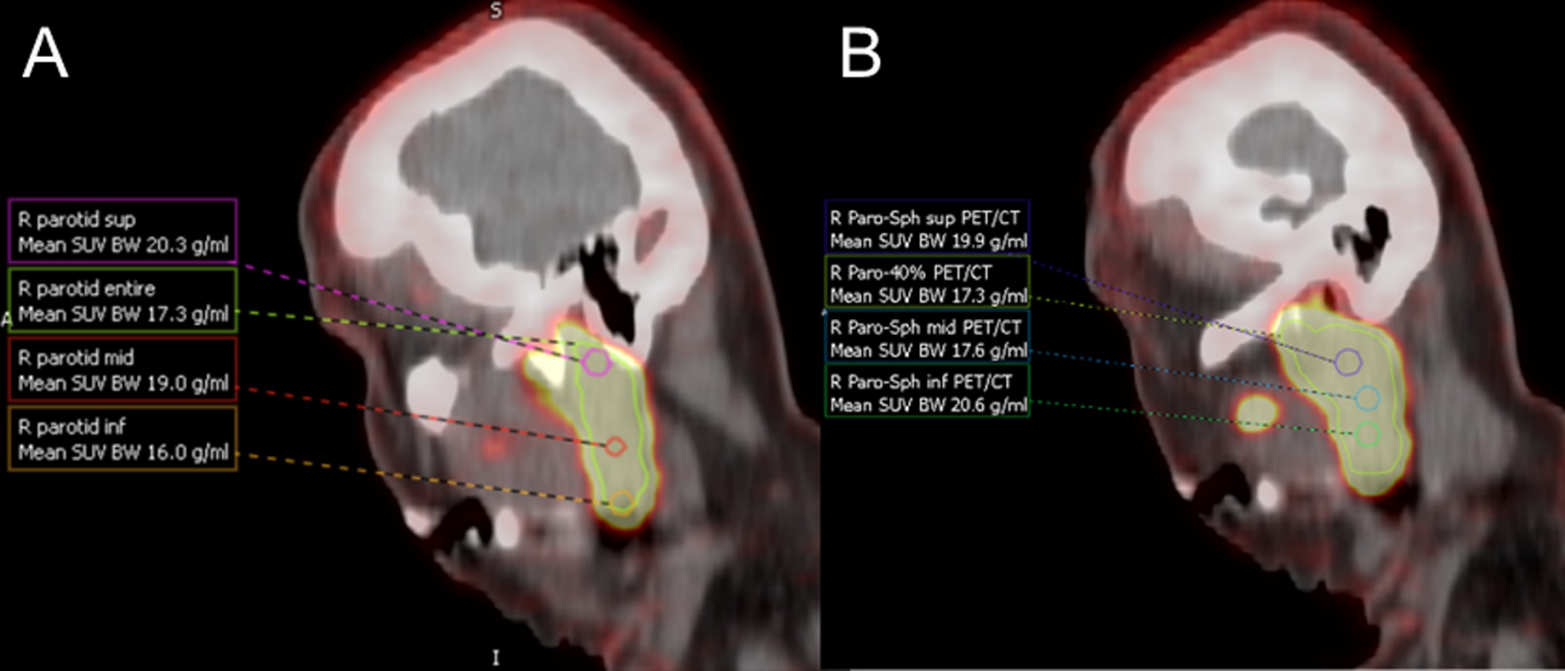
Example demonstrating variability for parotid region of interests (ROIs). Sagittal maximum intensity projection from the PET/MR [^18^F]DCFPyL PSMA tracer acquisition with labeled parotid gland and liver ROIs. Spatial variability of calculated SUV_mean_ for the 1-cm ROIs obtained from the superior (sup), middle (mid), and inferior (inf) right parotid gland is seen with values of 18.6, 22.4, and 21.0 g/mL respectively.

There was minimal inter-reader SUV_mean_ variability (bias±LOA) for blood pool (-0.13±0.42; 0.01±0.41), liver (-0.55±0.82; -0.22±1.3), or whole parotid gland (-0.05±0.31; 0.08±0.24) for wbPET/CT and wbPET/MR, respectively (Fig 1). Greater inter-reader variability was present for the 1-cm parotid gland ROIs for wbPET/CT and wbPET/MR respectively, in the superior (-1.75±5.49; -4.63±11.3), mid (-0.76±2.78; -1.05±3.86), and inferior (-1.42±5.26; -2.66±5.02) gland (Fig 2). Much of the inter-reader variability for the 1-cm parotid gland ROIs was likely due to spatial variability in parotid gland uptake (Fig 3).

### Inter-modality variability

**Fig 4:**
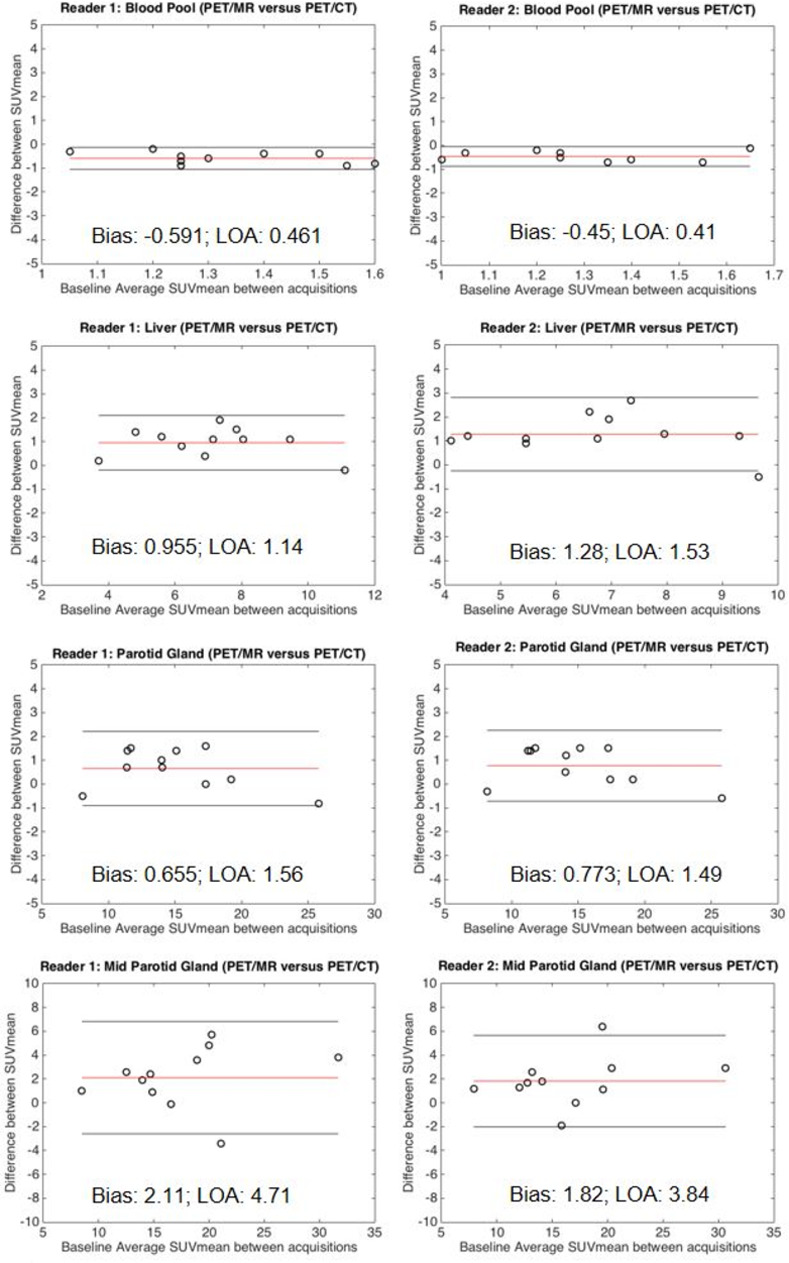
Bland Altman analysis assessing inter-modality variability between PET/CT and PET/MR acquisitions. A slight bias of decreased SUV_mean_ for blood pool (top) and increased SUV_mean_ for liver (middle) and parotid gland (bottom) was seen for wbPET/MR compared to wbPET/CT regardless of reader. The calculated bias (red line) as well as the upper and lower limits of agreement (outer black lines) are demarcated on each plot. The bias and limits of agreement are listed in the bottom-center.

When wbPET/CT was compared to wbPET/MR, there was a slight decrease in SUV_mean_ for blood pool (reader 1, -0.59 ± 0.46; reader 2, -0.45 ± 0.46) and slight increase for liver (reader 1, 0.96±1.1; reader 2, 1.3 ± 1.5) and whole parotid (reader 1, 0.66 ± 1.6; reader 2, 0.77 ± 1.5). However, the magnitude of overall bias was less than 1.5 in each case (Fig 4).

### Inter-subject variability

**Fig 5:**
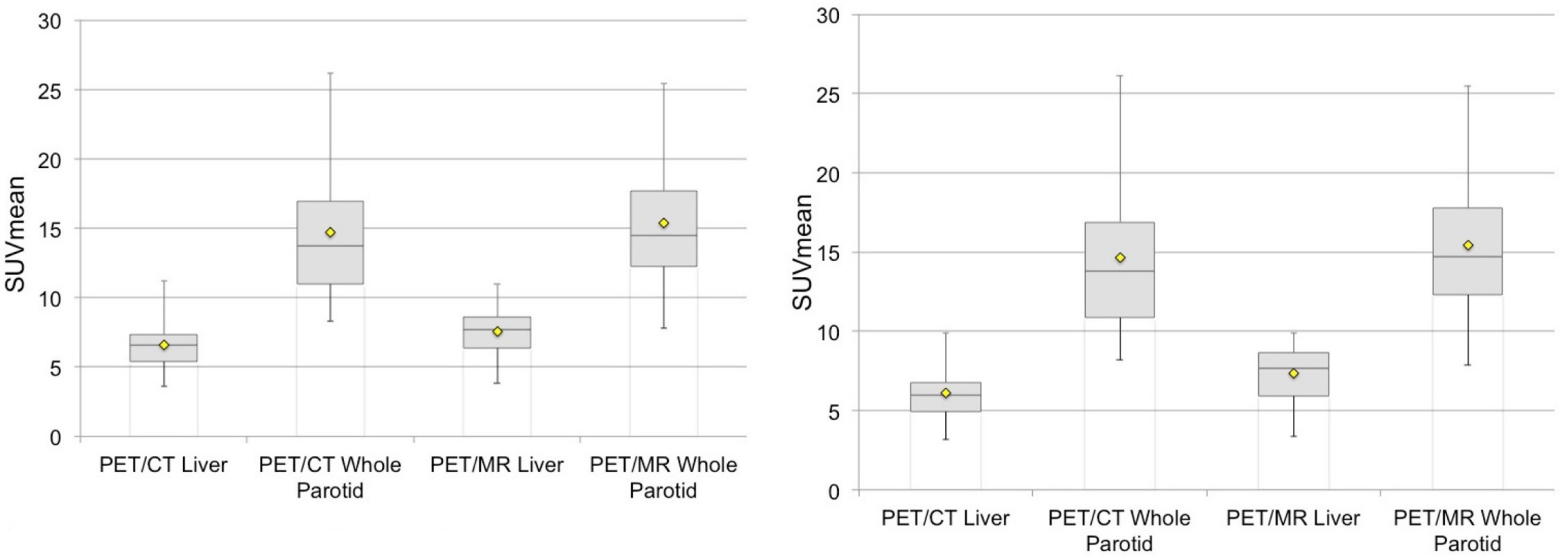
Box plots for SUV_mean_ of the liver and whole parotid gland regions of interest (ROIs) from both PyL PET/CT and PET/MR demonstrates greater interquartile and min-max range for the parotid compared to liver for both readers, reader 1 (A) and reader 2 (B) respectively. Box defines median and 1st and 3rd quartiles. Whiskers are maximum and minimum. Diamond defines the mean value.

The magnitude of variability was higher for the parotid gland, compared to liver and blood pool, regardless of modality or reader. When assessed using a percentage coefficient of variance, the difference between the liver and parotid variability was less divergent, although the CoV for liver SUV_mean_ ranged from 24–31% compared to 30–36%, 25–38%, 35–38%, and 33–45% for the whole, superior, mid, and inferior parotid gland measurements, respectively. Overall, blood pool had the lowest CoV, ranging from 15–21%. Inter-subject reference organ SUV_mean_ variability are detailed in Table 2 and highlighted in Fig 5.

**Table 2:** Reference organ inter-subject variability according to reader and modality.

	Reader 1			Reader 2		
	Median	IQR	CoV (%)	Median	IQR	CoV (%)
PET/CT						
Liver	6.7	1.8	31	6.0	2.4	30
Blood pool	1.6	0.2	16	1.6	0.3	15
Whole parotid	13.6	5.9	34	13.7	6.1	36
Superior Parotid	15.6	8.0	37	13.6	7.1	29
Mid Parotid	15.5	4.9	37	14.8	5.8	38
Inferior Parotid	13.7	4.8	45	12.8	7.0	44
PET/MR						
Liver	7.7	2.2	27	7.7	2.7	24
Blood pool	1.1	0.3	15	1.1	0.1	21
Whole parotid	14.5	5.5	31	14.7	5.5	30
Superior Parotid	17.4	6.4	38	12.8	4.3	25
Mid Parotid	16.5	6.5	35	15.0	6.9	36
Inferior Parotid	16.0	6.0	36	15.2	5.4	33

CoV, coefficient of variance; IQR, interquartile range.

## Discussion

This study sought to evaluate the variability of PSMA-based [^18^F]DCFPyL (PyL) PET quantitative reference standards. We found that liver, blood pool, and whole parotid gland quantitation show promise as reliable reference organs. Greater variability with 1-cm parotid ROIs may limit its use. Liver ROIs had less intra-subject variability compared to parotid SUV_mean_ which may be important to consider when establishing treatment or scoring cut-off values or thresholds.

Defining appropriate quantitative reference standards for PSMA-based PyL PET might allow for optimized interpretation of repeat studies and greater generalizability of research results. The PROMISE criteria proposed using a relative expression score with intermediate (score 2) activity defined as equal to or above liver but lower than parotid gland and high (score 3) activity defined as equal to or above parotid gland [33]. Qualitative evaluation using these reference organs was also included in the E-PSMA guidelines [26]. However, the use of quantitative reference organs as well as clinical evaluation based on the referenced guidelines may not be used regularly in clinical practice for many imaging centers. Still, the results of the current study certainly support the use of liver and blood pool uptake as quantitative parameters. Indeed, liver SUV_mean_ had minimal inter-reader variability with an absolute mean bias between readers of only 0.55 and 0.22 for PET/CT and PET/MR, respectively. The use of liver uptake for visual quantification of disease activity/score has been previously established, most notably through the Lugano criteria for Lymphoma [34]. More recently a phase-II trial evaluating [177Lu]PSMA-617 in the setting of metastatic prostate cancer used lesion 68Ga-PSMA-11 uptake that was significantly greater than liver [35]. Another study looking at the change in ^68^GA-PSMA in 43 patients treated systemically for metastatic castration resistant prostate cancer found a median change in SUV_max_ of -13.3% (IQR: -44 to 41%) [36].

Use of parotid gland uptake as a reference quantitative parameter is more nuanced. First, in the current study evaluation of 1-cm ROIs showed relatively high inter-reader variability (absolute mean bias from 0.76–4.63 and limits of agreement 2.78–11.3). This relatively high variability is most likely due to heterogenous expression throughout the parotid gland and secondary to variable blood flow. SUV_mean_ from a whole parotid ROI resulted in lower absolute mean bias (±limits of agreement) of 0.05 (±0.31) and 0.08 (±0.24) for reader 1 and 2, respectively. Therefore, if parotid uptake is used as a reference standard it might be prudent to use a whole gland ROI to minimize this variability. Similarly, if the parotid gland is utilized as a qualitative, or visual, reference standard [26] this may also minimize the effect of intra-gland heterogeneity.

Bland-Altman analysis and evaluation of repeatability is often best interpreted in relation to the clinical context under which it might be used. For example, Rowe et al. reported a median SUV_max_ of 7.4 (IQR: 4.2–12.9) for suspected osseous metastatic lesions that were ‘definitively’ or ‘equivocally’ positive on [^18^F]DCFPyL PET/CT [19]. Given the proximity of the average SUV_mean_ of liver reported in our study, compared to the reported median of osseous metastases, this might serve as an appropriate standard to evaluate for sites of possible metastatic disease.

When comparing modalities, blood pool SUV_mean_ was lower while liver and parotid gland were higher, although the absolute bias/increase was low (0.45–1.28) for the wbPET/MR compared to wbPET/CT. These differences were likely attributed to study design as the wbPET/MR was acquired approximately 120 minutes after the [^18^F]DCFPyL tracer injection whereas wbPET/CT was obtained 60 minutes after injection. This is supported by the work of Ferreira et al., which found a weak positive correlation between liver SUV_peak_ and [^18^F]DCFPyL uptake time [27]. A similar finding was seen in a study using [^68^GA]PSMA [37]. Randomization of acquisition order would be the ideal way to evaluate the effect of modality and scanner, not uptake time. In one study with [^68^GA]PSMA, an average of 20% higher SUV_max_ was calculated for PET/CT compared to same day PET/MR when the order was randomized [38]. Further work with randomization between timing of the PET/CT and PET/MR acquisition with the [^18^F]DCFPyL tracer may also be useful.

This study had important limitations. First, a single time point was utilized and thus test-retest repeatability cannot be assessed. Second, as discussed previously, differences in acquisition timing and detector characteristics confound the evaluation of wbPET/MR compared to wbPET/CT. Third, other features that can affect variation in reference organ quantification, such as physiologic conditions and uptake time, were not directly assessed in this study and future research in these areas may be useful. Finally, the overall study size was small and confirmation of the results of the study in future larger trials may be beneficial.

In conclusion, liver, blood pool, and whole parotid gland quantitation show promise as reliable reference normal organs for clinical and research PET applications. Variability with 1-cm parotid ROIs may limit its use.
